# Menetrier's Disease with Gastrointestinal Bleed

**DOI:** 10.4103/0972-9941.18999

**Published:** 2005-09

**Authors:** Kaushik Bhattacharya

**Affiliations:** Department of Surgery, Subham Hospital and Diagnostic Centre, Cooch Behar – 736101, West Bengal

**Figure 1:**
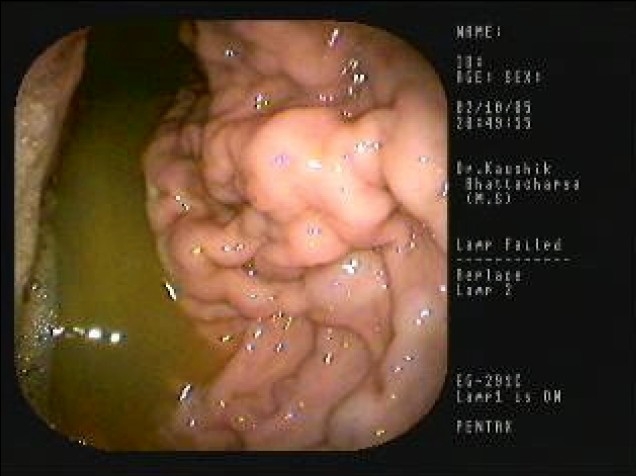
Menetrier's disease

**Figure 2:**
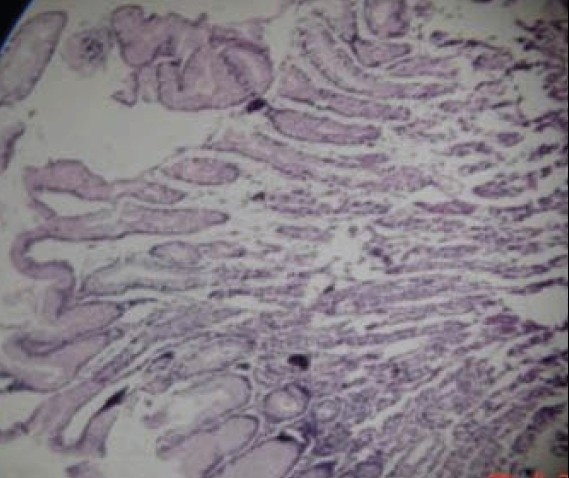
Histopathogical features of Menetrier's disease showing hyperplasia of foveolar cells

A 22-year-old male presented with recurrent upper gastrointestinal bleeding along with pain in the abdomen. An upper gastrointestinal endoscopy revealed Menetrier's disease affecting the body of the stomach, which was confirmed histopathologically [Figure 1]. There was hyperplasia of foveolar cells with elongation of the crypts, decrease in the number of gastric glands and mild lymphocytic infiltration within the lamina propria [Figure 2]. No endoscopic ultrasound was done in this patient due to its nonavailability. Menetrier's disease is a rare premalignant disorder of the stomach generally described as hypertrophic gastropathy associated with hypoproteinemia and achlorhydria. This disease is characterized by excessive mucous secretion, but differs little from normal stomach with regards to the types of mucin produced.[1] Gastric resection is still the most definitive treatment for the disease, but the appropriate extent of resection has not been determined.[2] The condition should be distinguished from hyper-rugosity of the gastric mucosa in which there is no protein and hyper-rugosity, which may be associated with Zollinger Ellison syndrome, malignancy, infectious diseases (cytomegalovirus, histoplasmosis, syphilis) and infiltrative diseases like sarcoidosis.
